# Synthesis and Antioxidant Activity of 7-Thio Derivatives of 6,7-Dihydro-1*H*-cyclopenta[*d*]pyrimidine-2,4(3*H*,5*H*)-dione

**DOI:** 10.3797/scipharm.1406-09

**Published:** 2014-10-29

**Authors:** Yuriy M. Kononevich, Ludmila S. Bobkova, Alexander S. Smolski, Anatoly M. Demchenko

**Affiliations:** 1 A. N. Nesmeyanov Institute of Organoelement Compounds, Russian Academy of Sciences, Vavilova str. 28, 119991, Moscow, Russian Federation; 2 Institute of Pharmacology and Toxicology, National Academy of Medical Science of Ukraine, Ezhena Pot’je str. 14, 03680, Kyiv, Ukraine; 3 T. G. Shevchenko Chernihiv National Pedagogical University, Het’mana Polubotka str. 53, 14013, Chernihiv, Ukraine

**Keywords:** 6,7-Dihydro-1*H*-cyclopenta[*d*]pyrimidine-2,4(3*H*,5*H*)-dione, Alkylation, Antioxidant activity, Fe^2+^-dependent oxidation of adrenaline

## Abstract

New 7-thio derivatives of 6,7-dihydro-1*H*-cyclopenta[*d*]pyrimidine-2,4(3*H*,5*H*)-dione have been synthesized by the reaction of 3-cyclohexyl-7-thio-6,7-dihydro-1*H*-cyclopenta[*d*]pyrimidine-2,4(3*H*,5*H*)-dione with alkylhalogenides. The synthesized compounds were tested for antioxidant activity on the model of Fe^2+^-dependent oxidation of adrenaline *in vitro*. It was found that the antiradical activity of 7-thio derivatives of 6,7-dihydro-1*H*-cyclopenta[*d*]pyrimidine-2,4(3*H*,5*H*)-dione significantly depends on the structure of the substituent which is part of the thioether fragment of the base molecule.

## Introduction

It is well-known that pyrimidine derivatives are a class of organic compounds which are actively investigated for the presence of pharmacological activity. A large number of highly effective synthetic drugs, based on pyrimidine derivatives, have been widely used. Pyrimidine derivatives condensed with a five-membered heterocyclic moiety occupy a special place among the large variety of biologically active derivatives of pyrimidine. Well-known drugs such as allopurinol, sildenafil, and tubercidin represent this type of bioactive pyrimidine.

In addition to the great interest in the derivatives of pyrimidine condensed with a heterocyclic moiety, a lot of attention has also been paid to the derivatives of pyrimidine condensed with an alicyclic moiety. Thus, it has been established that the derivatives of cycloalkyl[*d*]pyrimidine show hypoglycemic [1], antimalarial [2], analgesic [3], anti-inflammatory [4], antithrombin [5], PDE4 inhibitor [6], and other types of biological activity [7]. Earlier it was reported that the derivatives of cyclopenta[*d*]pyrimidine-2,4(3*H*,5*H*)-dione showed high spasmolytic activity [8–11].

Although derivatives of cycloalkyl[*d*]pyrimidine were studied for many types of pharmacological activities, the antioxidant activity of this class of compounds was not investigated. Antioxidants are compounds that inhibit oxidation reactions by neutralizing free radicals which are formed during these reactions. It is known that many diseases are associated with the oxidative damage of biomolecules [12, 13]. The use of antioxidants can prevent radical-induced damage and thus allow us to stave off and treat the diseases mentioned above.

In the continuation of our studies for the search of new biologically active compounds among the derivatives of cycloalkyl[*d*]pyrimidine, the synthesis of new 7-thio derivatives of 6,7-dihydro-1*H*-cyclopenta[*d*]pyrimidine-2,4(3*H*,5*H*)-dione has been carried out. For the synthesized compounds, the antioxidant activity was evaluated.

## Results and Discussion

### Chemistry

The 7-thio derivatives of 6,7-dihydro-1*H*-cyclopenta[*d*]pyrimidine-2,4(3*H*,5*H*)-dione **4a–h** were prepared in three steps from 6,7-dihydro-1*H*-cyclopenta[*d*]pyrimidine-2,4(3*H*,5*H*)-dione **1** using methods which we described earlier (Sch. 1) [10]. By the reaction of compound **1** with *N*-bromosuccinimide in acetic acid at room temperature, the 7-bromo derivative of 6,7-dihydro-1*H*-cyclopenta[*d*]pyrimidine-2,4(3*H*,5*H*)-dione **2** was prepared in good yield. The next stage was the synthesis of 7-thio 6,7-dihydro-1*H*-cyclopenta[*d*]pyrimidine-2,4(3*H*,5*H*)-dione **3** from compound **2** and thiourea. The reaction was carried out at reflux in ethanol followed by the treatment with aqueous sodium hydroxide. Acidification of the reaction mixture by HCl gave 7-thio 6,7-dihydro-1*H*-cyclopenta[*d*]pyrimidine-2,4(3*H*,5*H*)-dione **3** as a solid precipitate in good yield.

**Sch. 1:**
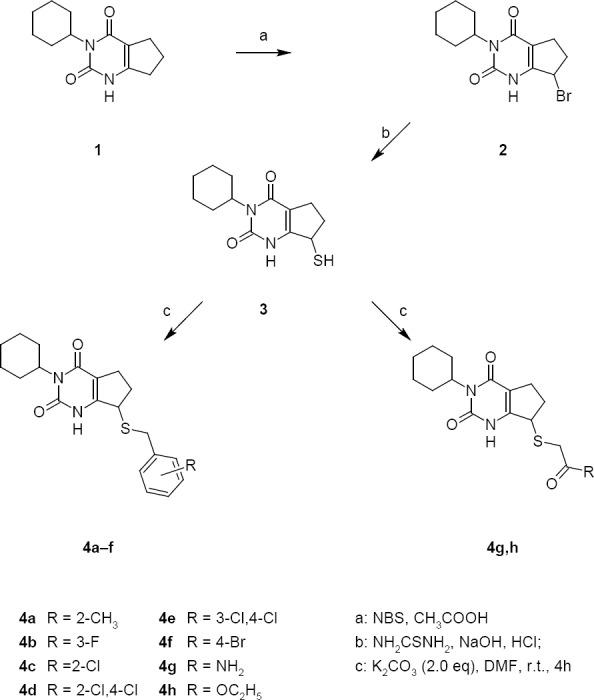
Synthesis of 7-thio derivatives of 6,7-dihydro-1*H*-cyclopenta[*d*]pyrimidine-2,4(3*H*,5*H*)-dione 4a–h

The alkylation of 7-thio 6,7-dihydro-1*H*-cyclopenta[d]pyrimidine-2,4(3*H*,5*H*)-dione **3** by various alkylhalogenides gave the series of 7-thio derivatives of 6,7-dihydro-1*H*-cyclopenta[*d*]pyrimidine-2,4(3*H*,5*H*)-dione **4a–h** which were tested for antioxidant activity. The reactions were carried out in dimethylformamide at room temperature with potassium carbonate as a base. The structures of synthesized compounds were confirmed by ^1^H- and ^13^C-NMR, IR spectroscopy, and mass spectrometry.

From the ^1^H-NMR spectra, it can be seen that there are signals of cyclohexyl and cyclopentyl fragments in the strong field of spectra in the form of multiplets. One of the protons of the cyclopentane fragment which is located near the sulfur atom can be seen as a doublet at 3.99–4.15 ppm with J=5.5–6.7 Hz. The position and the shape of the signal protons of the S-CH_2_ fragment essentially depends on the nature of the substituent in this fragment. Thus, in the case of the acetamide substituent, the signal of protons of the S-CH_2_ fragment is located at 3.29 ppm as a singlet. In the benzylic substituents, the signal of protons of the S-CH_2_ fragment is shifted towards the weak field and located at 3.8 ppm. It should also be noted that the presence of the halogen atom in the *ortho*-position of the benzyl fragment leads to the appearance of the signal of protons of the S-CH_2_ fragment as two doublets with J=12.2–14.0 Hz, that is typical for the AB type spin system. Amide protons are located in the weak field at 11.09-11.44 ppm as a broad singlet.

### Antioxidant Activity

The antioxidant activity of the 7-thio derivatives of 6,7-dihydro-1*H*-cyclopenta[*d*]pyrimidine-2,4(3*H*,5*H*)-dione was evaluated using the model of Fe^2+^-dependent oxidation of adrenaline *in vitro* (Table 1). 2,6-Di-*tert*-butyl-4-methylphenol (ionol), quercetin, ketorolac, and diclofenac were used as reference substances.

**Tab. 1:**
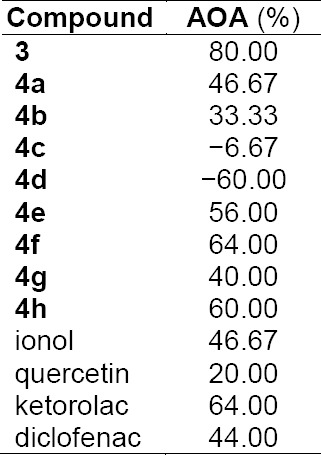
Antioxidant activity of 7-thio derivatives of 6,7-dihydro-1*H*-cyclopenta[*d*]pyrimidine-2,4(3*H*,5*H*)-dione 3, 4a–h in comparison with various antioxidants

It was established that compounds **3**, **4h**, **4a**, **4f**, and **4e** showed high antiradical activity that was even higher than the antiradical activity of the reference substances (ionol, quercetin, ketorolac, diclofenac). Thus, compound **3** that contains the SH group showed the highest antioxidant activity (80%) of all, which is not surprising since thiols are effective interceptors of free radicals [14]. Benzylthioethers that contain halogen atoms in the *para*-position and do not contain substituents in the *ortho*-position also showed high antioxidant activity (**4f** – 64%, **4e** – 56%). A significant antiradical activity was found for thio-derivatives **4h** (60%) and **4g** (40%) containing ethylacetate and acetamide fragments, respectively. The antiradical activity of compound **4a** – a compound containing an orthomethylbenzyl moiety is on par with the activity of ionol. It should also be noted that compound **4b**, which contains the metafluorobenzyl moiety, showed but moderate antioxidant activity (33.33%).

Despite the fact that the majority of 7-thio derivatives of 6,7-dihydro-1*H*-cyclopenta[*d*]pyrimidine-2,4(3*H*,5*H*)-dione showed high antioxidant activity, there are compounds in this series that enhance the oxidation of adrenaline and thus show prooxidant activity. These compounds are orthohalogenated benzylthioethers **4c** (−6.67%) and **4d** (−60%).

Thus, the antiradical activity of 7-thio derivatives of 6,7-dihydro-1*H*-cyclopenta[*d*]pyrimidine-2,4(3*H*,5*H*)-dione, which was evaluated by the model of Fe^2+^-dependent *in vitro* oxidation of adrenaline, significantly depends on the structure of the substituent attached to the thioether fragment of the base molecule.

## Experimental

### Materials and Methods

All solvents were purified before use. *N*-bromosuccinimide, thiourea, 2-methylbenzyl chloride, 3-fluorobenzyl chloride, 2-chlorobenzyl chloride, 2,4-dichlorobenzyl chloride, 3,4-dichlorobenzyl chloride, 4-bromobenzyl chloride, 2-chloroacetamide, and ethyl chloroacetate were purchased from Acros Organics and used without purification. Compounds **2**, **3**, **4b**, and **4h** were prepared using methods described previously [10]. The reactions were monitored by thin-layer chromatography (TLC) using Fluka silica gel (60 F 254) plates (0.25 mm). Visualization was made with UV light. The melting points of the synthesized compounds were taken on a melting point tube. Infrared spectra were recorded on a Bruker Tensor 37 spectrometer (Germany). The ^1^H-NMR spectra were recorded on a Bruker WP 250 SY spectrometer (250.13 MHz) (Germany). Chemical shifts are reported relative to chloroform (δ=7.25 ppm) for ^1^H-NMR. The mass spectra were recorded on an Agilent LC/MSD SL 1100 instrument (USA).

#### 3-Cyclohexyl-7-sulfanyl-6,7-dihydro-1H-cyclopenta[d]pyrimidine-2,4(3H,5H)-dione (3)

The mixture of bromide **2** (0.0064 mol) and thiourea (0.0064 mol) in ethanol (20 mL) was stirred with reflux for 4 h. After the reaction was complete, the crystalline solid was filtered off and added to an aqueous solution of sodium hydroxide (30 mL, 5%). The mixture was heated to boiling. After the reaction mixture was cooled to room temperature and acidified with HCl, the thiol **3** was obtained as a colorless solid. The solid product was filtered off and washed with water. Yield 54%, Crystalline solid, mp. 168–170°C. IR (KBr), ν, cm^−1^: 3105, 2937, 2856, 1707, 1645, 1530,1403. ^1^H-NMR (DMSO-d_6_) δ 1.15 (m, 1H, Cy), 1.28 (m, 2H, Cy), 1.51 (d, 2H, *J* = 12.1 Hz, Cy), 1.63 (d, 1H, *J* = 12.1 Hz, Cy), 1.79 (m, 3H, Cy, -CH_2_CH_2_CH-), 2.35 (m, 2H, Cy) 2.48 (m, 2H, -CH_2_CH_2_CH-), 2.62 (m, 1H, -CH_2_CH_2_CH-), 3.34 (s, 1H, SH), 4.13 (s, 1H, S-CH), 4.64 (m, 1H, N-CH), 11.15 (s, 1H, NH). ^13^C-NMR (DMSO-d_6_) δ 25.0, 25.4, 25.9, 28.0, 28.1, 32.3, 51.9, 108.4, 151.9, 154.6, 161.3. MS *m/z*: 267.20 [(M+H)^+^].

### General Procedure for the Synthesis of 7-thio Derivatives of 6,7-dihydro-1H-cyclopenta[d]pyrimidine-2,4(3H,5H)-dione (4a–h)

The mixture of thiol **3** (0.002 mol), alkylhalogenide (0.002 mol), and K_2_CO_3_ (0.004 mol) in DMF (5 mL) was stirred at room temperature for 4 h. After the reaction was complete, the mixture was poured into water (25 mL). The solid product was filtered off and washed with water. The residual product was purified by recrystallization from isopropanol.

#### 3-Cyclohexyl-7-[(2-methylbenzyl)sulfanyl]-6,7-dihydro-1H-cyclopenta[d]pyrimidine-2,4(3H,5H)-dione (4a)

Yield 81%, Crystalline solid, mp. 86–88°C. IR (KBr), ν, cm^−1^: 2930, 2855, 1707, 1639, 1520, 1423. ^1^H-NMR (DMSO-d_6_) δ 1.07–1.34 (m, 3H, Cy), 1.47 (d, 2H, *J* = 12.2 Hz, Cy), 1.61 (d, 1H, *J* = 12.2 Hz, Cy), 1.77 (d, 2H, *J* = 12.2 Hz, Cy), 2.04 (m, 1H, -CH_2_CH_2_CH-), 2.23-2.58 (m, 8H, -CH_2_CH_2_CH-, Cy, CH_3_), 3.71 (d, 1H, *J* = 12.2 Hz, S-CH_2_), 3.86 (d, 1H, *J* = 12.2 Hz, S-CH_2_), 4.05 (d, 1H, *J* = 6.7 Hz, S-CH), 4.60 (m, 1H, N-CH), 7.05-7.22 (m, 4H, C_6_H_4_), 11.31 (s, 1H, NH). ^13^C-NMR (DMSO-d_6_) δ 18.6, 25.1, 26.0, 28.1, 28.2, 30.4, 31.8, 47.2, 49.8, 110.3, 125.9, 127.4, 129.7, 130.4, 135.3, 136.5, 152.1, 152.2, 161.4. MS *m/z*: 371.15 [(M+H)^+^].

#### 3-Cyclohexyl-7-[(2-chlorobenzyl)sulfanyl]-6,7-dihydro-1H-cyclopenta[d]pyrimidine-2,4(3H,5H)-dione (4c)

Yield 61%, Crystalline solid, mp. 88–90°C. IR (KBr), ν, cm^−1^: 3204, 2930, 2864, 1709, 1666, 1628. ^1^H-NMR (DMSO-d_6_) δ 1.02–1.33 (m, 3H, Cy), 1.46 (d, 2H, *J* = 12.2 Hz, Cy), 1.61 (d, 1H, *J* = 12.8 Hz, Cy), 1.76 (d, 2H, *J* = 12.8 Hz, Cy), 2.01 (m, 1H, -CH_2_CH_2_CH-), 2.23-2.56 (m, 5H, -CH_2_CH_2_CH-, Cy), 3.84 (d, 1H, *J* = 12.8 Hz, S-CH_2_), 3.94 (d, 1H, *J* = 13.4 Hz, S-CH_2_), 4.07 (d, 1H, *J* = 6.1 Hz, S-CH), 4.59 (m, 1H, N-CH), 7.23-7.30 (m, 2H, C_6_H_4_), 7.39-7.45 (m, 2H, C_6_H_4_), 11.23 (s, 1H, NH). ^13^C-NMR (DMSO-d_6_) δ 25.1, 26.0, 28.1, 28.2. 30.4, 31.2, 47.2, 52.0, 110.4, 127.3, 129.1, 129.5, 131.2, 133.1, 135.5, 151.9, 152.0, 161.3. MS *m/z*: 391.10 [(M+H)^+^].

#### 3-Cyclohexyl-7-[(2,4-dichlorobenzyl)sulfanyl]-6,7-dihydro-1H-cyclopenta[d]pyrimidine-2,4(3H,5H)-dione (4d)

Yield 79%, Crystalline solid, mp. 89–91°C. IR (KBr), ν, cm^−1^: 2932, 2855, 1703, 1663, 1639, 1520, 1472, 1422. ^1^H-NMR (DMSO-d_6_) δ 1.03-1.33 (m, 3H, Cy), 1.45 (d, 2H, *J* = 11.6 Hz, Cy), 1.60 (d, 1H, *J* = 11.6 Hz, Cy), 1.76 (d, 2H, *J* = 12.2 CH, Cy), 2.00 (m, 1H, -CH_2_CH_2_CH-), 2.22-2.56 (m, 5H, -CH_2_CH_2_CH-, Cy), 3.85 (d, 1H, *J* = 14.0 Hz, S-CH_2_), 3.91 (d, 1H, *J* = 14.0 Hz, S-CH_2_), 4.05 (d, 1H, *J* = 6.1 Hz, S-CH), 4.57 (m, 1H, N-CH), 7.34 (dd, 1H, *J_1_* = 2.4 Hz, *J_2_* = 8.6 Hz, C_6_H_3_), 7.49 (d, 1H, *J* = 7.9 Hz, C_6_H_3_), 7.58 (d, 1H, *J* = 2.4 Hz, C_6_H_3_), 11.23 (s, 1H, NH). ^13^C-NMR (DMSO-d_6_) δ 25.0, 26.0, 28.0, 28.1, 30.3, 30.6, 47.0, 52.0, 110.3, 127.3, 128.8, 132.2, 132.5, 133.9, 134.8, 151.9, 152.0, 161.2. MS *m/z*: 425.00 [(M+H)^+^].

#### 3-Cyclohexyl-7-[(3,4-dichlorobenzyl)sulfanyl]-6,7-dihydro-1H-cyclopenta[d]pyrimidine-2,4(3H,5H)-dione (4e)

Yield 81%, Crystalline solid, mp. 90–92°C. IR (KBr), ν, cm^−1^: 2932, 2855, 1707, 1639, 1520, 1423. ^1^H-NMR (DMSO-d_6_) δ 1.06-1.32 (m, 3H, Cy), 1.43 (d, 2H, *J* = 11.6 Hz, Cy), 1.60 (d, 1H, *J* = 11.6 CH, Cy), 1.76 (d, 2H, *J* = 12.8 Hz, Cy), 1.96-2.09 (m, 1H, -CH_2_CH_2_CH-), 2.19-2.56 (m, 5H, -CH_2_CH_2_CH-, Cy), 3.85 (s, 2H, S-CH_2_), 3.99 (m, 1H, S-CH), 4.53 (m, 1H, N-CH), 7.31 (dd, 1H, *J_1_* = 7.9 Hz, *J_2_* = 1.8 Hz, C_6_H_3_), 7.5 (d, 1H, *J* = 8.6 Hz, C_6_H_3_), 7.6 (d, 1H, *J* = 1.8 Hz, C_6_H_3_), 11.20 (s, 1H, NH). ^13^C-NMR (DMSO-d_6_) δ 25.1, 26.0, 28.0, 28.1, 30.0, 32.3, 46.5, 51.9, 110.1, 128.9, 129.4, 130.2, 130.5, 130.8, 139.6, 151.9, 152.0, 161.0. MS *m/z*: 425.00 [(M+H)^+^].

#### 7-[(4-Bromobenzyl)sulfanyl]-3-cyclohexyl-6,7-dihydro-1H-cyclopenta[d]pyrimidine-2,4(3H,5H)-dione (4f)

Yield 74%, Crystalline solid, mp. 190–192°C. IR (KBr), ν, cm^−1^: 2932, 2855, 1705, 1639, 1520, 1425. ^1^H-NMR (DMSO-d_6_) δ 1.04-1.32 (m, 3H, Cy), 1.45 (d, 2H, *J* = 11.6 Hz, Cy), 1.60 (d, 1H, *J* = 12.8 Hz), 1.76 (d, 2H, *J* = 11.6 Hz, Cy), 1.94-2.09 (m, 1H, -CH_2_CH_2_CH-), 2.21-2.55 (m, 5H, -CH_2_CH_2_CH-, Cy), 3.82 (s, 2H, S-CH_2_), 4.00 (d, 1H, *J* = 5.5 Hz, S-CH), 4.54 (m, 1H, N-CH), 7.28 (d, 2H, *J* = 7.9 Hz, C_6_H_4_), 7.44 (d, 2H, *J* = 8.6 Hz, C_6_H_4_), 11.09 (s, 1H, NH). ^13^C-NMR (DMSO-d_6_) δ 25.1, 26.0, 28.0, 28.1, 30.1, 33.0, 46.7, 51.9, 110.1, 120.0, 130.8, 131.0, 137.6, 152.0, 152.1, 161.1. MS *m/z*: 435.05 [(M+H)^+^].

#### 2-[(3-Cyclohexyl-2,4-dioxo-2,3,4,5,6,7-hexahydro-1H-cyclopenta[d]pyrimidin-7-yl)sulfanyl]acetamide (4g)

Yield 69%, Crystalline solid, mp. 183–185°C. IR (KBr), ν, cm^−1^: 3393, 3179, 2934, 2857, 1705, 1653, 1429. ^1^H-NMR (DMSO-d_6_) δ 1.02-1.32 (m, 3H, Cy), 1.48 (d, 2H, *J* = 11.6 Hz, Cy), 1.61 (d, 1H, *J* = 12.2 Hz, Cy), 1.76 (d, 2H, *J* = 12.2 Hz, Cy), 1.88-1.99 (m, 1H, -CH_2_CH_2_CH-), 2.25-2.57 (m, 5H, -CH_2_CH_2_CH-, Cy), 3.29 (s, 2H, S-CH_2_), 4.15 (m, 1H, S-CH), 4.61 (m, 1H, N-CH), 7.31 (s, 1H, NH_2_), 7.67 (s, 1H, NH_2_), 11.44 (s, 1H, NH). ^13^C-NMR (DMSO-d_6_) δ 25.1, 25.7, 26.0, 28.1, 30.7, 34.5, 48.0, 52.1, 110.0, 152.0, 152,7, 161.5, 171.7. MS *m/z*: 324.05 [(M+H)^+^].

### Antioxidant Activity

The antioxidant activity of the 7-thio derivatives of 6,7-dihydro-1*H*-cyclopenta[*d*]pyrimidine-2,4(3*H*,5*H*)-dione was estimated by the reaction of Fe^2+^-dependent oxidation of adrenaline. The incubation mixture contained 2.5 mL of a carbonate buffer (0.2 M, pH=10.6), 50 µL of a 0.1% solution of adrenaline, and 50 µL of the test compound. The mixture was thoroughly stirred and quickly placed into the SF-46 spectrophotometer. The optical density was measured at 347 nm during 20 min [15]. Into the control probe the carbonate buffer, solution of adrenaline, solution of FeSO_4_, and 50 µL DMSO were added. The mathematical processing of the results was carried out by the methods of variational statistics using the Student’s t-test [16].

## Authors’ Statement

### Competing Interests

The authors declare no conflict of interest.
